# Biological Role of MYCN in Medulloblastoma: Novel Therapeutic Opportunities and Challenges Ahead

**DOI:** 10.3389/fonc.2021.694320

**Published:** 2021-06-14

**Authors:** Sumana Shrestha, Alaide Morcavallo, Chiara Gorrini, Louis Chesler

**Affiliations:** ^1^ Division of Clinical Studies, Institute of Cancer Research (ICR), London and Royal Marsden NHS Trust, Sutton, United Kingdom; ^2^ Division of Cancer Therapeutics, The Institute of Cancer Research (ICR), and The Royal Marsden NHS Trust, Sutton, United Kingdom

**Keywords:** MYCN, medulloblastoma, targeted therapy, metabolism, immunotherapy, PROTACs

## Abstract

The constitutive and dysregulated expression of the transcription factor MYCN has a central role in the pathogenesis of the paediatric brain tumour medulloblastoma, with an increased expression of this oncogene correlating with a worse prognosis. Consequently, the genomic and functional alterations of MYCN represent a major therapeutic target to attenuate tumour growth in medulloblastoma. This review will provide a comprehensive synopsis of the biological role of MYCN and its family components, their interaction with distinct signalling pathways, and the implications of this network in medulloblastoma development. We will then summarise the current toolbox for targeting MYCN and highlight novel therapeutic avenues that have the potential to results in better-tailored clinical treatments.

## Introduction

The MYC family of transcription factors, including c-MYC (MYC), MYCL and MYCN are amongst the most commonly altered genes in cancer, including paediatric cancers (1). Tumorigenic activity of the MYC family can result from constitutive activation of associated mitogenic signalling pathways e.g., Wingless (WNT), Hedgehog (SHH), Transforming growth factor beta (TGF-β), or through direct genetic alterations from amplification or chromosomal aberrations. Sequence homology between the two proteins, MYC and MYCN remain relatively high, and similarities remain between organisation of loci, and protein binding sites (2–4). Pioneering developmental studies have been integral in illustrating the interchangeable nature of the MYC proteins, in particular between MYC and MYCN (5). These studies assessing the phenotypic consequences of *Myc* or *Mycn* deficiency in mouse development identified expression divergence during the early developmental stages. Whilst null homozygosity for both *Myc* and *Mycn* resulted in embryonic lethality (approximately E10.5-E11.5), *Myc*-null embryos were associated with marked reduction in size and a general delay in primitive development of the heart (6). *Mycn*-null embryos also exhibited delayed development and stunted growth, as well as diminished cellularity in organs that normally express abundant *Mycn* transcripts, most notably the cranial and spinal ganglia (7–9). Significantly, despite a compensatory *Myc* increase observed in *Mycn* deficient embryos (8), developmental defects occurred that suggested a unique and essential role for *Mycn* during CNS development. Conversely, replacement of endogenous *Myc-*coding sequences with *Mycn-*coding sequences showed that Mycn is capable of performing most of the essential functions of Myc required for embryonic development and proliferation (5). Whilst the proteins share similarities in structure and binding partner MAX (10), the differences remain in their spatial and temporal expression patterns, with MYCN showing a preference to the early hindbrain development (11–14). Overall, both proteins at the transcriptional level can orchestrate the cell cycle machinery and stimulate cell growth, division, and regulate the differentiation states of cells throughout development.

In this review, we will focus on the role of the MYC family proteins, specifically MYCN, in different subgroups of the childhood brain tumour medulloblastoma (MB). MYC proteins play an important role in MB biology and often are dysregulated in all MB tumours, with *MYC, MYCN* and *MYCL1* each showing commitment to specific subgroup (15). *MYC* and *MYCN* amplifications especially are prominent in MB due to the highly aggressive nature of tumours associated with these aberrations (16). *MYC* and *MYCN* have been considered undruggable for many years as they carry out essential functions in proliferative tissues, suggesting that their inhibition could cause severe side effects. Only recently has there been an improvement in making their protein surfaces amenable to binding small molecules, further accelerating their use in therapeutics (17). We will highlight the potential application of several new therapeutic strategies targeting MYCN and its signalling partners to tackle the overarching obstacles.

## Medulloblastoma

### Clinical and Molecular Diversity of Medulloblastoma

Medulloblastoma is one of the most prevalent malignant paediatric brain tumours (WHO grade IV) (18). MB arises from the posterior fossa and features a heterogeneous tumour landscape. MB accounts for ~63% of childhood intracranial embryonal tumours, and has an incidence of 4.9 per 1 million children, peaking at ~7 years of age (19, 20). The tumour is usually proximal to the fourth ventricle, making metastasis through cerebrospinal fluid (CSF) flow common (21). The current standard of care consists of maximal surgical resection followed by cranio-spinal irradiation (CSI) in patients >3 years, and multi-agent chemotherapy. The overall survival rate ranges from 40-90%, depending on the molecular subtype and other factors such as extent of dissemination and degree of resection. Whilst survival rates have improved overtime due to better understanding and implementation of CSI, ~1/3 of patients still succumb to the disease, and survivors often experience debilitating neurologic, endocrinologic, and cognitive sequelae from treatment (22).

### Different Subgroups and Subtypes

Initially, using gene expression analysis techniques, MB was segregated into four distinct molecular subgroups, with differences in genomic drivers, mutational events, methylation patterns and clinical characteristics (23). These groups are known as: wingless (WNT), Sonic hedgehog (SHH), and, group 3 (Grp3), and group 4 (Grp4), also known as non-WNT/non-SHH (24–27). Due to the well-defined developmental pathways of WNT and SHH, many studies have been able to dissect the mechanism of these two groups, whereas Grp3 and Grp4 have recently raised attention owing to next-generation sequencing techniques (15, 28–31). Further gene expression and DNA-methylation analysis of the subgroups by Cavalli et al. introduced additions layers of heterogeneity within the four main subgroups, these are as follows: WNT; WNT-α and WNT-β, SHH; SHH-α, SHH-β, SHH-χ, SHH-δ, Group 3; Grp3-α, Grp3-β, Grp3-χ and Group 4; Grp4-α, Grp4-β, Grp4-χ (32). Adding to this, other variations of subtypes emerged during the same time as several research groups utilised different sample analysis methods (33, 34). Whilst the use of separate analytic techniques provides a more dynamic and richer dataset, it is now essential to compare and combine these differences to produce a single, streamline set of subtypes.

Comparing the clinical features of the subgroups, the WNT subgroup has the most favourable clinical outcomes, with the overall survival standing at >90% (35). However, this is the least common MB subtype, accounting for only 10% of MBs (23). The favourable prognosis associated with activation of the WNT signalling pathway is now being exploited for other subgroups of this cancer (36). The majority of these tumours (86%) harbour activating mutations in β-catenin (*CTNNB1*), a central orchestrator of the canonical WNT pathway (33, 37, 38), or mutations in the tumour suppressor gene *APC (71%)* (39). Further prominent genes identified from whole genome sequencing include *DDX3X (7.6%)*, *SMARCA4 (3.4%)*, *TP53 (~10%)* and *KMT2D (~7%)* (40).

The SHH-subgroup, despite its heterogeneity, is the best clinically and molecularly characterised MB-subgroup. The age of the patient is especially important here as each age group has a distinct transcriptomic profile; adult patients (SHH-δ) show frequent mutation in the TERT promoter, whilst younger patients show enrichment in focal amplifications of *MYCN*, *GLI2*, and *YAP1*, frequent germline or somatic TP53 mutations, and more recently discovery of germline variants in *ELP1* (32, 41–44). *ELP1* encodes the scaffold protein elongator complex protein 1 (ELP1) which is involved in neuronal migration and is responsible for transcriptional elongation (45, 46). Furthermore, *PTCH1* mutation is highly frequent in this subgroup, the only distinction of this within the subtypes is the number of additional aberrations accommodating this mutation; with a higher aberrational load seen in the adult subtype (SHH-δ). More recent research shows a prominent role for *TP53* dysregulation. This frequently arises in conjunction with chromothripsis, a catastrophic genomic rearrangement commonly occurring due to micronucleus formations (29, 42, 47).

In contrast to WNT and SHH, both Grp3, and Grp4 have very few prominent driver genes. Nonetheless, they both show distinct genetic events which define each subgroup as separate entities. Grp3 MB primarily occurs during infancy and childhood and is associated with a high rate of disseminated disease. This subgroup is defined by high levels of *MYC* amplification and a particular genetic signature related to increased transcription and translation (48). Important genes dysregulated at a somatic level include *SMARCA4*, *KBTBD4*, and *KMT2D*. Furthermore, recent studies have shown increased activation of genes representing the Notch and TGFβ signalling pathways, and a particular inclination for activation of GFI1/GFI1B through enhancer hijacking (49). Grp4 MB is the most prevalent MB subgroup. With a lack of single gene mutations, this subgroup features a higher frequency of disposition to somatic mutations, with notable mutations in histone-modifying genes such as *KDM6A*, *ZMYM3* and *KMT2C*. The recent discovery of ERBB4-SRC signalling in Grp4 tumours has highlighted this pathway as a hallmark of Grp4 MB (50). Both Grp3 and Grp4 groups have recurrent mutations in *KBTBD4*, underpinning the gene as a common candidate tumour driver (33).

Cancer predisposition syndromes remain a risk factors for the development of MB and account for approximately 5-6% of MBs (39). Germline mutations in WNT signalling pathway genes such as *APC* mutations, found in Turcot Syndrome, can lead to the WNT subgroup. SHH MB can be initiated through various germline mutations such as *PTCH1*, occurring in the autosomal dominant condition Gorlin syndrome (known as nevoid basal cell carcinoma syndrome), or aberration in germline *TP53* as seen in Li-Fraumeni syndrome (33). Furthermore, germline mutation in *SMO* from Curry-Jones syndrome is also associated with SHH MB (39). With the advancement in stem cell research and patient-derived iPSC culture systems, this information will inevitably allow for more accurate modelling and prediction of the development of particular subgroups of MB (51).

The innate differences within this highly heterogeneous cancer provides an insight into the putative cells of origin residing in different regions of the cerebellum (32, 52). The embryonic nature of MB makes it difficult to pinpoint the exact cell of origin. Therefore, it is essential to investigate the complete genetic aetiology of these tumours in order to build more accurate and robust models of the disease. The normal development of the cerebellum serves as a healthy control for the development of MB. MB cells are thought to arise from progenitor cell populations from early hindbrain development. This has been investigated for WNT and SHH MB, with WNT tumours thought to arise from the extracerebellar lower rhombic lip, and SHH from cerebellar granule cell precursors (GCPs) (53). More recently, it was postulated that for Grp4, the cellular origin consisted of more differentiated neuronal population, with glutamatergic cells including residues of unipolar brush cells and glutamatergic cerebellar nuclei (30, 54). Whilst for Grp3 origin remains quite vague, with studies referring to a potential origin of undifferentiated progenitor-like lineage with high MYC activity (54).

## The Role of MYCN in the Origin of Medulloblastoma

### MYCN in Cerebellar Development

When looking at the development of MB, the environment in which the tumour grows should be treated almost as a crime scene, as Bailey and Cushing once wrote "the histogenesis of the brain furnishes the indispensable background for an understanding of its tumours'' (55). A defect in the normal expansion of the cerebellar precursor population can lead to uncontrolled proliferation, resulting in the development of MB.

**Figure 1:**
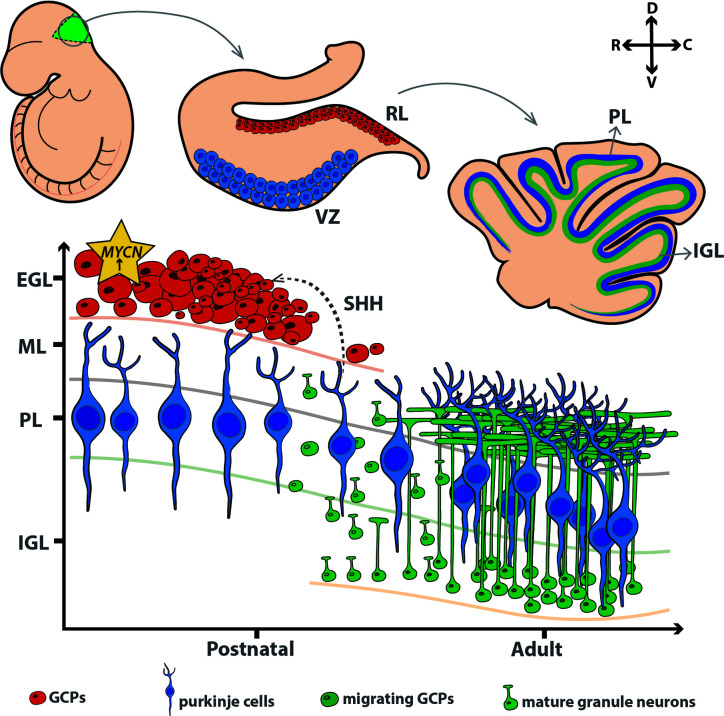
MYCN maintains the proliferation of granule cell progenitors in the external granule layer during early development. VZ, ventricular zone; RL, rhombic lip; PL, purkinje layer; IGL, internal granule layer; EGL, external granule layer; ML, molecular layer; GCP, granule cell precursors. D, dorsal; C, caudal; V, ventral; R, rostral.

The developing cerebellum is moulded by three distinct pools of progenitor cells; these consist of GCPs from the deep nuclei (emerging from rhombic lip), GABAergic Purkinje cells (arising from multipotent precursors of the primary germinal epithelium in the roof of the 4th ventricle), and CD133/Nestin+ cells (the white matter of the postnatal cerebellum). During postnatal development, GCPs rapidly proliferate and expand in response to SHH secreted by Purkinje cells (56), and mature to become cerebellar granule neurons – the largest neuronal population in the brain (57), as shown in **Figure 1**.

*MYCN* plays a fundamental role in orchestrating both normal and abnormal development of the cerebellum, with critical functions in precursor growth and maturation **(** **Figure 1** **)**. MYCN is present at low levels in many neonatal tissues and expressed at particularly high levels in the hindbrain (8, 58). *MYCN* expression persists during differentiation stages where *MYC* is downregulated (8). *MYCN* expression is predominant in neural stem cells and progenitor cell populations, with its expression diminishing after the cells become committed to more differentiated states. One putative mechanism employed by MYCN to sustain CNS development is the conservation of large domains of chromatin in an euchromatic state (59). This is demonstrated through double knockout animal models of *MYC* and *MYCN*, which showed gene alterations in chromatin structure (60).

MYCN also contributes to cerebellum development downstream of SHH. SHH is an extracellular signalling molecule with a critical role in regulating growth and differentiation in the developing brain (61, 62). SHH signalling upregulates MYCN through activation of PI3K, a corollary of this is glycogen synthase kinase 3 beta (GSK-3β) inhibition, preventing destabilisation of the MYCN protein by GSK-3β and halting skp-cullin-F-box (SCF)-FBXW7 induced proteasomal degradation (63). Several studies have demonstrated SHH as a primary driver of the expansion of GCPs through direct upregulation of MYCN, highlighting the importance of *MYCN* in the proliferation of GCP cells and for their responses to SHH (64, 65). As a mitogen, SHH induces genes involved in cell cycle progression and DNA replication, mainly during the period of post-natal hindbrain expansion. In parallel, the role of *MYCN* is most critical during this phase. Dysfunctional *MYCN* can prime the progenitor cells by altering internal regulatory mechanisms, making them more susceptible to defects in cerebellar development. Studies have shown *MYCN* null neural precursors have high levels of specific cyclin dependent kinase inhibitors (CDKI), p18I^nk4c^ and p27^KIP1^ which induces differentiation programmes in cells, supported by reduced levels of cyclin-D2 (66, 67). More specific to its structure, preventing phosphorylation of the MYCN amino-terminal impedes cell cycle exit of GCPs (68); phosphorylation of the S62 priming site of MYCN by CDK1/cyclin A/B prevents this continuous activation of the cell cycle and allows cell cycle exit (69). MYCN has a vital role in the early cerebellum development. By orchestrating a time-dependent expansion of progenitor cells to form the EGL, it indicates a window of high activity, after which it is downregulates to allow cell cycle arrest and subsequent differentiation and maturation of the cells.

### The Role of MYCN in MB Groups

Collectively, this family of oncogene is of particular interest as *MYC* and *MYCN* are each committed to specific subtypes of MB. MYC is often found to be overexpressed in WNT tumours, despite a lack of *MYC* amplification, whereas amplification of MYC is commonly detected in G3 tumours (31). Both *MYCN* amplification and overexpression is observed in SHH MB. *MYCN* amplifications are also present in G4 tumours, however, this is generally at a much lower level compared with the SHH subgroup (31). *MYC* and *MYCN* amplifications in particular have been the main focus in MB due to the highly aggressive nature of tumours associated with these aberrations (16). Alongside genetic abnormalities, dysregulated epigenetic modifiers are also frequently observed in more aggressive medulloblastoma tumours, including those harbouring *MYCN*/MYCN abnormalities (70, 71). The preference of MYC to a distinct subtype suggests potential ideas about MB tumorigenesis.

### The WNT Group

Moderately high levels of MYCN and MYCL1 are observed in this subtype compared to Grp3 and Grp4 (72). Furthermore, *MYC* is also highly expressed with comparable levels to those seen in Grp3 (13). This high level *MYC* expression could be explained by *MYC* being a downstream target of WNT signalling (73). Whilst MYC expression usually correlates with poor prognosis in other MB subgroups, the WNT subtype displays the most positive prognosis within the subgroups, regardless of MYC levels (74).

### The SHH Group

Gene amplification is a very common event in SHH MB. The most prevalent amplifications include *MYCN* and *MYCL1*, as well as other important genes such as *GLI2*, *MDM4*, *PPM1D* and *YAP1* (15, 75). Patients with SHH subtype MB have a frequent gain of chromosome 2, which harbours *MYCN*, this may also explain the resulting *MYCN* amplification event seen regularly in this subgroup. In subtypes where *MYCN* amplification co-occurs with *TP53*-mutations, there is a worsening of the overall outcome (32, 76). An event linking the two together is chromothripsis. Tumours with high levels of this complex genome arrangement show a positive correlation in the frequency of MYC/MYCN amplifications (77). The chronological order of *TP53* mutation, *MYCN* amplification and chromothripsis is largely unknown and yet to be explored. Pursuing this further will inevitably shed light on novel DNA repair mechanisms which can be utilised for therapeutic targeting.

Studies have shown that MYCN has dual-capacity to produce either SHH-dependent (63) or SHH-independent MB (78). Formation of either is highly dependent on the temporal expression of *MYCN*, either during embryonic or postnatal development (58). This further highlights MYCN's dynamic role in CNS development. Novel pathways fuelling MB growth include the evolutionarily conserved signalling pathway known as the Salvador-Warts-Hippo (Hippo) pathway (79). SHH signalling may have cross talk with the Hippo pathway to regulate important downstream efforts such as the Yes-associated protein (YAP), an oncoprotein shown to promote proliferation of CGPs (80). Indeed, genomic profiling of OLIG2-expressing glial progenitors as transit amplifying cells of SHH-MB revealed that these cells activate oncogenic networks including HIPPO-YAP/TAZ and AURORA-A/MYCN (81).

### Group 3

The majority of Grp3 tumours are characterised by high protein levels of MYC, either induced by *MYC* amplifications or by aberrant MYC expression (41). Differential analysis of super-enhancers has identified MYC as a prominent target in Grp3. Thus, MYC is noted as the key driver of Grp3 MB (30). Plasmacytoma Variant Translocation 1 (PVT1) gene fusion in Grp3 is linked to chromothripsis and MYC amplification on chromosome 8q24 (82). New molecular stratification of this subtype into different subgroups, has shown that subgroup II and III harbour amplifications of the *MYC* oncogene and are associated with poor outcomes (83). Interestingly, subgroup V, characterised by amplification of both *MYC* and *MYCN*, is associated with moderate clinical outcomes (83). Moreover, the increased abundance of many proteins involved in mRNA processing, transcription and translation observed in Grp3 MB is associated with high *MYC* expression (48, 50). Interestingly, whilst this subgroup is mainly associated with MYC amplifications, MYCN amplifications is also seen in a minority of patients (5%) (33).

### Group 4

The most frequent somatic copy-number alterations (SCNAs) in this group target the gene *SNCAIP* (synuclein, alpha interacting protein) (15). While MYCN amplification also occur in this subtype, they are mutually exclusive with SNCAIP duplications (31).

### Current Management of MYCN-Associated MB

Since the identification and isolation of MB as a distinct entity in 1926 by Cushing and Bailey, the prognosis of patients has alleviated from no survival to now, the most positive outcome of 80% 5-year overall survival (OS). This improvement was led through continuous progression in understanding the biological mechanisms behind this cancer and strengthened by emerging technologies and treatments. Although this OS sounds very positive, the reality of the age of these patients, coupled with the harsh quality of life (QOL) observed after the treatments (84, 85) pushes this scientific field to develop more novel and targeted therapies which can ameliorate the dismal QOL.

While our understanding of MB biology and molecular features has greatly improved over the last decade, current treatment regimens for MB have been relatively unchanged. These strategies are principally tailored based on clinico-radiological risk criterion, used to define the standard-risk (SR) or high-risk (HR) group (86). Children who are >3 years with no evidence of metastatic disease (M0), post-surgical residual tumour <1.5 cm^2^, and histologically non-anaplastic are categorized as SR, while the remaining are considered HR. Children >3 years who have significant residual disease following surgery, large cell/anaplastic (LC/A) histology and metastatic disease have a worse prognosis with poor survival outcome (87).

Medulloblastomas are typically more radiosensitive than other paediatric brain tumours, including glioblastomas (GBM). Therefore, radiotherapy is an essential element in the multidisciplinary management of children with MB, and postoperative craniospinal axis radiotherapy is considered a curative treatment. Commonly, children >3 years, receive surgery, external beam radiation to the spine and brain, combined with multidrug chemotherapy (cisplatin, vincristine, and cyclophosphamide). While both SR and HR children are treated with radiation, HR patients are given larger boosts of radiation. Children <3 years are treated postoperatively with high-dose chemotherapy as an irradiation-avoiding strategy or with non-high-dose chemotherapy during induction followed by a reduced dose of conformal radiotherapy (CRT) to the tumour bed (88, 89). Radiation is generally avoided in children <3 years due to the adverse effects on the developing brain. Radiation in older children has been linked to reduced IQ and induction of secondary cancers, vasculopathy, hearing loss, and future strokes (90–92). The standardised treatment of MB solely based on histopathology and clinico-radiological risk stratification can lead to unpredictable relapses and therapeutic failures. Disease relapse is the most adverse prognostic factor in MB, occurring in approximately 30% of patients (93). Children with tumour relapse receive various strategies, including continuous administration of low doses of chemotherapeutic, high-dose chemotherapy, intrathecal medication, and re-irradiation, but these approaches are commonly unsuccessful (94, 95).

It is evident from the review that MYCN is a very attractive therapeutic target. Nevertheless, it has proven challenging to target, with current techniques unsuccessful in exploiting the molecule for therapeutic gain (10). Structurally, MYC proteins lack any enzymatic activity/globular functional domains, which makes it unapproachable for structure-based virtual screening, and undesirable for the long-established enzyme inhibitor design (96). Adding to this, MYCN is also known as an intrinsically disordered (ID) oncoprotein, meaning the protein structure of MYCN in isolation fails to adopt a defined three-dimensional structure (97). This is advantageous for its role as a transcription factor as the ID structure allows MYCN to hold a larger surface area for increased interaction with numerous other proteins – the disordered domains mean it can be "re-used" in multiple pathways (98). However, this makes it challenging to target MYCN directly.

Further to its structure and function, the widespread expression of *MYCN* by all early proliferating cells also poses a concern. The numerous target genes of MYCN makes it difficult to define critical oncogenic effector pathways for precise drugging. Thus, targeting this oncogene may present with unacceptable toxicities (99). However, as most "normal" CNS cells spend the majority of their life in quiescence, the adverse effects may be more negligible than expected (100). Limited direct targeting of MYCN has motivated strategies to look at indirect or MYCN-dependent interactions instead. Due to the high growth-inducing activity of MYCN, its mechanism is controlled at multiple steps.

## Emerging Therapeutic Opportunities

### Targeting MYCN Stability

The stability of the MYCN protein itself is a critical level of regulation. MYCN is controlled by phosphorylation of specific residues, most of which takes place within Myc box I (MBI). First, phosphorylation mediated by CDK1-CyclinA/B1 complexes occurs at S62, which permits recognition. Second, phosphorylation activity occurs via serine/threonine kinase GSK3β on T58. The phosphorylating activity of GSK3β causes degradation of MYCN. MYCN protein has an ephemeral half-life (20-30 minutes), and is tightly regulated by E3 ubiquitin ligase (E3 ligase) through recruitment and proteasomal degradation (97, 101). Whilst there is also a role played by calpains for the turnover of MYC, the majority of degradation is carried out by the ubiquitin-proteasome system (UPS) (102, 103). The ligases FBXW7 and TRUSS have essential roles in restricting MYCN functions via the UPS. FBXW7 recognises MYCN upon phosphorylation at both S62 and T58, causing MYCN to be specifically degraded during mitosis, providing a mechanism which induces cell cycle exit and differentiation of neural progenitor cells. Thus, increasing the level of FBXW7 would be especially attractive to MB with *MYCN* overexpression. More recently, in a study by Skowron et al. looking at the transcriptome of 250 human SHH MB, they discovered missense mutation within the tryptophan-aspartic acid motif (WD40) of *FBXW7* in SHH MBs (42). This supports the idea of targeting this important ligase to alter MYCN activity. Another ligase responsible for restricting MYCN function is the HECT (Homologous to the E6-AP Carboxy Terminus)-domain ubiquitin ligase, HUWE1. This degradation system acts by priming the protein through addition of Lys 48-mediated linkages. HUWE1 carries this out for both MYC and MYCN, but shows a greater efficiency for the latter (104). Interfering with this mechanism, at the MYCN protein level, remains a potential strategy of intervention.

**Figure 2:**
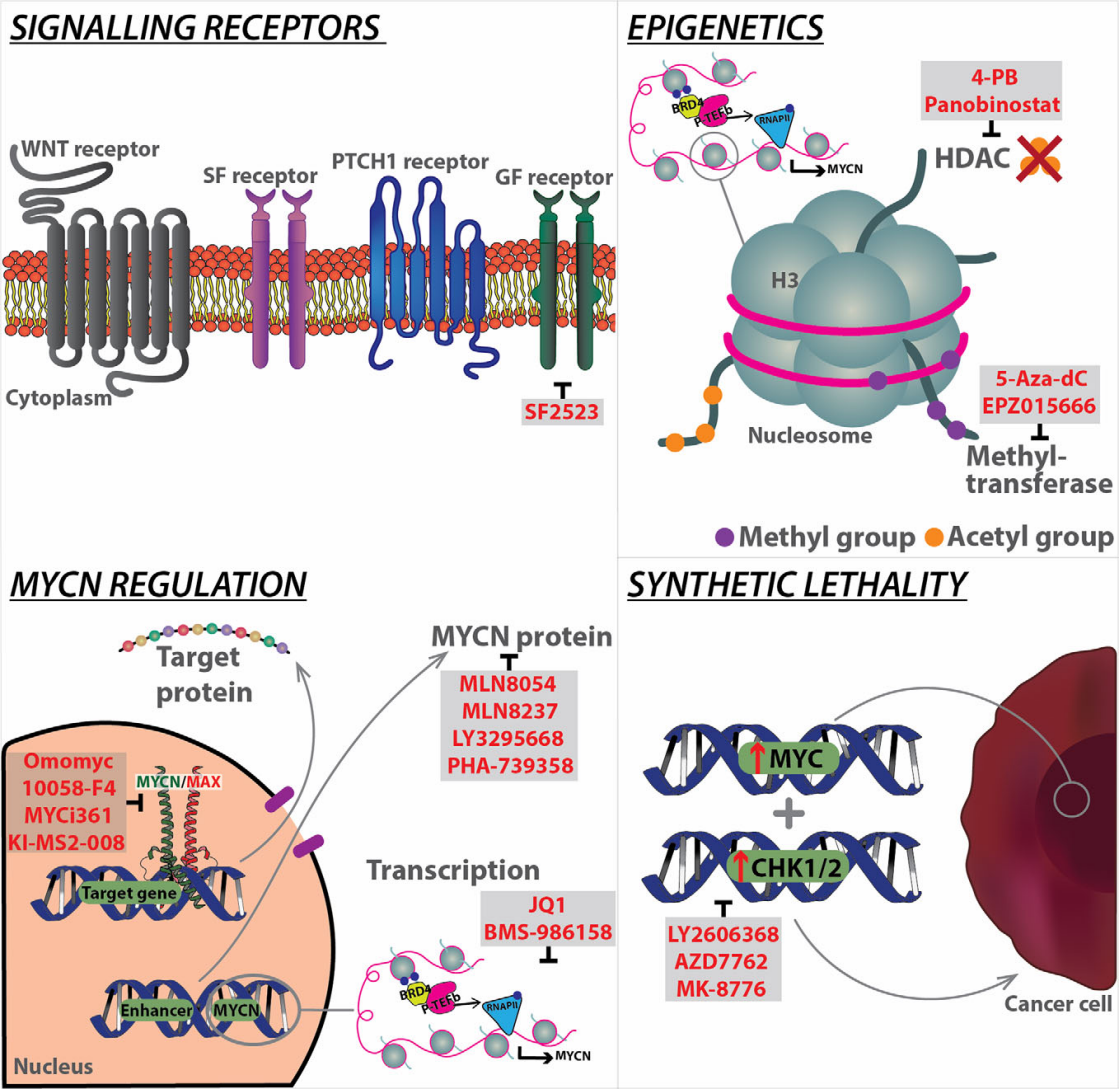
Overview of current strategies targeting MYCN at different levels, from signalling receptors to downstream regulation, as well as epigenetics, and synthetic lethality. Specific modalities (highlighted in red) have been developed to target the mechanistic components of each pathway. BRD4, Bromodomain-containing protein 4; GF, Growth Factor; HDAC, Histone deacetylases; MAX, MYC-associated factor X; P-TEFb, Positive transcription elongation factor b; PTCH1, Patched 1; RNA pol II, RNA polymerase II; SF, Survival factor; WNT, Wingless.

The degradation of MYCN can be further inhibited by the activation of PI3K/AKT/mTOR axis, as active AKT can phosphorylate and inactivate GSK-3β, leading to MYCN stabilisation. Targeting PI3K may therefore be valuable to control the level of *MYCN* (105) **(** **Figure 2** **)**. This has been attempted through various candidate inhibitors such as taselisib, copanlisib, pictilisib, buparlisib, dacotilisib and idelasib, only to find the emergence of resistance to be common, and usually associated with upregulation of *MYCN*. To overcome this, studies have tried to utilise a combination therapy method with compounds such as SF2523 (106). This compound was investigated in a study by Andrews et al. in which it was able to inhibit both the MYC transcriptional co-factor, BRD4 and PI3K with increased efficacy and reduced toxicity to animals (106). More recent compounds targeting this pathway, and are ongoing clinical trials include LY3023414 [NCT03155620] and AZD2014 [NCT02813135]. In addition to the PI3K/AKT/mTOR pathway, WNT and SHH also play a role in inactivating GSK-3β, leading to upregulation and stabilisation of MYCN. Both pathways hold potential targets to regulate this stabilisation.

Recently, Aurora kinase A (AURKA), a member of the Aurora family of mitotic regulators, has been shown to form a complex with MYCN, to prevent its degradation by FBXW7. AURKA stabilises MYCN via a direct interaction with a protein binding site flanking MYCN's MBI sequence (107), this stabilisation of MYCN exacerbates its oncogenic functions, and prevents differentiation of neuroblasts in MYCN-driven neuroblastoma (NB) cell lines, leading to aberrant proliferation (108). It is likely that AURKA exerts similarly deleterious effects in MYCN-driven MB, with evidence for this provided by significantly decreased tumour volumes, and a tendency towards increased survival in *Ptch1^+/-^;p53^-/-^* mice treated with the AURKA inhibitor CD535 (109). Other inhibitors which target the AURKA complex with MYCN include MLN8054 and MLN8237. Upon destabilisation of the complex by these small molecule inhibitors, AURKA is no longer able to protect MYCN from proteasomal degradation. Using MLN8237, which blocks the interaction between AURKA and MYCN, our group was able to demonstrate that AURKA inhibition is effective against NB in a MYCN-driven transgenic mouse model (TH-MYCN), in which high-level expression of MYCN is driven in neural crest by a tyrosine hydroxylase (TH) promoter (110). Correlating with this finding, MLN8237 also significantly impaired the growth of MB allografts derived from GTML (Glt1-tTA/TRE-MYCN-Luc) tumour-derived neurosphere cell lines (111, 112). Additionally, MLN8237's *in vivo* activity was positively confirmed using a panel of human NB xenografts (113). Another AURKA inhibitor, namely PHA-739358, suppress proliferation of human SHH MB models, including allografts of *Patched* mutant tumour cells and patient-derived xenografts (114) **(** **Figure 2** **)**.

These findings have led to the investigation of AURKA inhibitors for the treatment of MYCN-dependent paediatric cancers. However, despite the encouraging results in pre-clinical studies, clinical trials with different Aurora Kinase inhibitors showed a limited efficacy against solid tumours (115). This has been attributed to mechanisms of resistance triggered by strong upregulation of ATP-binding cassette transporters, such as ABCB1, ABCG2 and ABCC2, and the emergence of AURKA mutations, impairing the efficient binding of the inhibitor in the ATP pocket of the enzyme and functional single nucleotide polymorphisms (SNP) (116, 117). In an analysis of a highly specific AURKA inhibitor LY3295668 in 560 cancer cell lines, NB was among the most sensitive tumour type tested, with *MYC/MYCN* amplification identified as among the strongest predictors of sensitivity to this agent (**Figure 2** **)**. Phase I trial of alisertib with irinotecan and temozolomide showed promising results prompting to a phase 2 study in children with relapsed/refractory NB. While these clinical studies supported a potential role for AURKA inhibition in the management of patients with advanced NB, patients with MYCN/MYC-driven tumours still showed poor outcomes despite treatment with this regimen (118). Inhibition of another component of AURK family, AURKB, has been found to sensitize MYC overexpressed Grp3 MB cells to cell death both *in vitro* and *in vivo* (119).

### Targeting MYCN Transcriptional Activity

Another therapeutic opportunity against MYCN-dependent MB is the use of drugs that effect the transcriptional activity of MYCN. For instance, MYC family gene expression depends on the activity of the co-factor bromodomain and extra-terminal (BET) family member BRD4. The bromodomain and extra-terminal (BET) family contain bromodomains (BRD), acetyl-lysine-specific protein interaction modules that play a key role in regulating gene transcription and are evolutionarily conserved and present in diverse nuclear proteins (120).

The BET family member BRD4 is of particular relevance to MYC-driven MB **(** **Figure 2** **)**. *MYC* gene expression is dependent on the activity of the BET family members (121). BRD4 preferentially binds to acetylated lysine residues K9/14 of histone H3, and deacetylated lysine residues K5/K12 of histone H4 (122, 123), following this BRD4 interacts with the positive transcription elongation factor b (P-TEFb) complex, leading to RNA polymerase II transcriptional activity (124). BRD4 is recruited to a wide range of promoter regions, including those for G1 cell cycle regulators (125, 126), and MYC (121, 127, 128), following this BRD4 co-recruits P-TEFb leading to gene transcription, which in the case of MYC is essential for MYC-dependent stimulation of its target genes (129). Taken together this highlights the potential of BET proteins, in particular BRD4, in driving cell cycle and MYC dysregulation, a corollary of which may be aberrant proliferation and tumorigenesis. By using a cell permeable BET inhibitor (BETi) called JQ1, a thieno-triazolo-1,4-diazepine, which displaces BET bromodomains from chromatin by competitively binding to the acetyl lysine recognition pocket, different research teams have demonstrated that tumours with deregulated MYC are susceptible to JQ1 inhibition both *in vitro* and *in vivo* (121, 127, 130, 131). In an unbiased screen of a collection of 673 genetically characterized tumour-derived cellular models, NB cell lines were identified as among the most JQ1 sensitive and MYCN amplification as the most predictive marker of sensitivity (132). Additional studies have demonstrated that JQ1 also suppresses MYC/MYCN expression and MYC/MYCN-associated transcriptional activity in MB, resulting in an overall decrease in MB cell viability (132–134). JQ1 treatment has been shown to be effective in MYC- and MYCN-driven MB by targeting cancer dependency genes driven by super-enhancers. More recently, the pan-BETi clinical compound Molibresib (GSK525762) shows positive outcome in Phase I and awaits further clinical trial result (135) (**Figure 2** **)**.

Cyclin-dependent kinases, especially CDK1 and CDK2, are key players in stabilizing phosphorylation of MYC proteins at S62 upon activation (69, 136, 137). Mechanistically, the response to BET inhibitors in MB is regulated by the suppression of genes involved in neuronal differentiation and progression through the cell cycle. In particular, the upregulation of the cell-cycle regulator CCND2 is a key mediator of sensitivity or resistance to BET inhibitors. Indeed, cells that tolerate BET inhibition do not terminally differentiate, maintain high expression of CCND2, that allows them to cycle through the S-phase. More recently it has been shown that JQ1 combined with Milciclib, an inhibitor of the MYC-stabilising enzyme CDK2, results in synergistic anti-tumoral effects. Mice xenograft of the human MB MB002 cell line showed prolonged survival when treated with JQ1 and Milciclib compared to vehicle and individual JQ1 or Milciclib treatment (138). This provides a strategy by which MYC, an 'undruggable' protein, may be indirectly targeted for therapeutic gain. Several small molecule BET inhibitors, structurally related to JQ1, are in clinical development and have shown preliminary clinical activity in solid tumours and blood cancers (139, 140). A phase I clinical trial with the BET inhibitor, BMS-986158, is currently ongoing in patients with paediatric cancers [NCT03936465].

Cyclin dependent kinases such as CDK7 and CDK9 play a key role in regulating transcriptional activity of MYCN (141–143). We have identified strong enrichment for CDK9 at both the MYCN promoter and the distal super enhancer and shown that pharmacologic blockade of CDK9 using Fadraciclib targeted MYCN-dependent transcriptional landscape (143). Several small molecule inhibitors targeting CDK7 and CDK9 (such as fadraciclib, dinaciclib and BAY1143572) have been shown to inhibit MYCN transcription and selectively kill MYCN amplified or expressing neuroblastoma, medulloblastoma and other cancer cells and are currently in early phase clinical trial.

### Targeting MYCN-Associated Epigenetic Molecules

MYC family genes also regulate transcription via epigenetic modifications, suggesting that epigenetic drugs could be used in the clinic to successfully treat MYC/MYCN-amplified tumours. Epigenetic alterations and aberrant expression of genes controlling epigenetic mechanisms have been identified in several cancers, including NB and MB. In this regard, numerous *in vitro* and *in vivo* evidence indicate that histone deacetylase inhibitors (HDACi) suppress MYCN expression and are promising candidates for novel treatment strategies of paediatric cancers (144–146). Selective inhibition of HDAC8 by small-molecule inhibitors kills tumour growth in xenograft mouse models of MYCN-amplified NB (147). The combination of the HDAC inhibitor, 4-phenylbutyrate (4-PB) and the demethylation agent, 5-Aza-2'deoxycytidine (5-Aza-dC) reduces DNA methyltransferase activity, global methylation and induces apoptosis in MB cell lines (148). Ecker and colleagues found HDAC2 to be overexpressed in MB subgroups with poor prognosis (SHH, Grp3 and Grp4) harbouring a MYC amplification compared to normal brain and the WNT subgroup. Indeed, increased sensitivity to HDACi is specifically observed in MYC amplified cells (149). HDACi further enhances the anticancer efficacy of other therapeutic regimens, such as ionizing radiation (IR) and can synergize with PI3K or MAPK/ERK inhibitors to impair tumour growth in vivo (150–152). Mechanistically, HDACi have been associated with different biological activities in MB, including the dissipation of mitochondrial membrane potential, changes in cell stemness, increased expression of the FOXO1 tumour suppressor gene, enhancing mitochondrial apoptosis in a p53-dependent manner and inhibition of the Hedgehog signalling (150, 152–154). Taken together, these data provide strong support for clinical testing of HDACi in the treatment of paediatric brain cancer patients, particularly those with MB. Further studies supporting this include a phase I trial and pharmacokinetic study of SAHA in children with solid tumours found to be well-tolerated (155), and a phase-I consortium clinical study recommending vorinostat in combination with the proteasome inhibitor bortezomib for future phase 2 studies in children with recurrent or refractory solid tumours (156).

HDACs represent an important epigenetic mechanism by which MYCN exerts its transcriptional effects. Treatment of murine and human PDX medulloblastoma cell lines with the pan-HDAC inhibitor Panobinostat lead to significant decreases in cell viability, with the lowest IC50 (14.4nM) seen in Grp3 MYC-driven PDX cells, and a marginally higher IC50 (25.27nM) in Grp4 MYCN-driven PDX (152). Exploration of the mechanism of HDAC inhibition using mouse MYC-driven medulloblastoma cell lines and Grp3 MYC-driven PDX cells revealed that HDAC inhibition significantly alters gene expression in treated MB cells. Having particularly notable effects on BRD4 target genes, MYC target genes, and stem cell proliferation genes, which were all downregulated following Panobinostat treatment. Further analysis of differential gene expression changes in these cells identified increased FOXO1 expression, and its subsequent interactions, as a key driver of the efficacy of HDAC inhibitors in MYC-driven medulloblastoma cells. This increase in FOXO1 expression may also be synergistically increased via combination therapy of Panobinostat with the PI3K inhibitor buparlisib (BKM-120) (152). Whilst the potential efficacy of HDAC inhibitors such as Panobinostat has been relatively neglected in MYCN-driven Grp4 and SHH MB, data from studies of MYCN-driven NB may provide evidence for HDAC inhibitors having similar effects on MYCN activity to those seen in MYC-driven cell lines. In one study of particular note, Panobinostat and the BRD4 inhibitor JQ1 acted synergistically to increase apoptosis and inhibit growth in human Kelly and SK-N-BE (2) MYCN-driven NB cells, whilst also synergistically reducing MYCN protein levels, but not mRNA levels (157). Together these studies in Grp3 MB and NB suggest that inhibition of HDACs may also be efficacious in MYCN-driven medulloblastoma. Future studies utilising Grp4 MB PDX cells will be required to confirm this hypothesis (**Figure 2** **)**.

MYC-driven primary medulloblastoma tumours have high expression of the arginine methyltransferase PRMT5 compared to non-MYC medulloblastoma tumours and adjacent normal tissues (158). PRMT5 is the major symmetric arginine methylase of histone tails and this histone modification is associated to both transcription activation and repression (159). PRMT5-mediated arginine methylation modulates a variety of cellular processes including cell growth, metastasis, ribosome biogenesis, cellular differentiation, gene transcription, germ cell specification, alternative splicing, and Golgi apparatus formation. Interestingly, the PRMT5 inhibitor EPZ015666 significantly suppressed cell growth and induced apoptosis in MYC-driven medulloblastoma cells (159) (**Figure 2** **)**. A variety of PRMT5 enzymatic inhibitors are currently applied in clinical trials of myelodysplastic syndrome, acute myeloid leukaemia, breast cancer and B cell non-Hodgkin lymphoma, prompting for further investigation in medulloblastoma [NCT03614728, NCT03573310, NCT02783300] (160).

### Targeting MYC-MAX Complexes

The bHLH-LZ structure of MYCN allows it to dimerise with various proteins, this is particularly relevant for its obligate partner MAX, with this interaction forming a stable four-helix bundle. MYCN/MAX heterodimers are required for non-consensus binding, as well as binding to the E-box sequences (161). Once bound to promoters of target genes, the complex can recruit transcriptional coactivators, elongation factors, and histone modifying enzymes to initiate gene transcription. Antagonists of this heterodimer complex represent strong candidates for MYCN-specific inhibitors. MAX forms homodimers, and heterodimers with other partners such as MNT and the MAD family members MAD1-4; these compete with MYCN for MAX. Complexes with MAD predominantly occur in resting or differentiated cells, whilst MYCN/MAX complexes are common in proliferating cells (162, 163). A potent inhibitor of the complex is Omomyc. This is a dominant-negative Myc peptide which facilitates the binding with the MYC protein through four specifically designed amino acid substitutions, thus disrupting the binding between MYC/MAX. This molecule has shown to promote apoptosis is many cancers retaining high MYC activity (133, 164), in particular with a preference for MB tumours (165). Other notable small-molecule MYCN/MAX inhibitors include 10058-F4, MYCi361, MYCi975 and KI-MS2-008 (17, 166, 167) (**Figure 2** **)**.

### Synthetic Lethal Targets of MYCN

A revolutionary method of indirectly targeting MYCN is to use the approach of synthetic lethal interactions. This term is defined as the extreme form of negative genetic interaction wherein the combination of two genes leads to cell death, whilst the two genes alone have no effect on viability of the cell. Synthetic lethal screens for MYCN amplification/overexpression have been more extensively investigated in NB, and more recently in MB due to identification of specific cell cycle checkpoint kinases (Chk1/2). In particular for Grp3 MB, Endersby et al. showed increased sensitivity of MYC amplified Grp3 MB cells to these check inhibitors (Chki) Prexasertib (LY2606368, (Chk1/2i), AZD7762 (Chk1/2i), and MK-8776 (Chk1i), with LY2606368 showing superior activity over the other compounds (**Figure 2** **)**. Ongoing basket trial for this compound is further investigating its anti-tumour activity [NCT02873975]. Furthermore, when used in combination with typical cancer drugs, LY2606368/gemcitabine combination showed specific activity for Grp3 MB subgroup alone. Whilst SHH MB showed reduced sensitivity to the LY2606368 compound alone, better outcome was seen when used in combination as LY2606368/cyclophosphamide (168). Current ongoing trial for these combinations include SHH and Grp3/4 patients [NCT04023669].

## Future Areas of Research for Innovative Therapies

### MYCN-Driven Cancer Metabolism

Being a high-grade tumour, MB must balance energy metabolism with the need to synthesize the macromolecules essential for its rapid proliferation. This contrasts with lower grade tumours that do not require constant accumulation of biomass and can therefore prioritize ATP production. Likewise, the neural progenitors from which it derives, MB cellular metabolism is characterized by increased lipogenesis and aerobic glycolysis. Indeed, both normal and malignant neuronal cells face similar challenges: they need the largest amount of ATP to support electrical activity and intercellular communication, but this requirement must be in balance with the additional metabolic requirements of rapid proliferation (169).

During the early stage of development, the rapid expansion of cerebellar GCPs (CGCP), fuelled by SHH signalling, compete for intermediates for the synthesis of lipids, nucleic acids and proteins with the downstream generation of ATP. SHH induces lipogenesis in CGCPs through a mechanism dependent on E2F1 transcriptional activity, involving the up-regulation of fatty acid synthase (FASN) and acetyl-CoA carboxylase 1 (ACC1). In parallel, it down-regulates fatty acid catabolism enzymes, including acyl-CoA oxidase 1 (ACOX1) and medium chain acyl-CoA dehydrogenase (MCAD) (170–172). SHH signalling also induces aerobic glycolysis in CGCPs and tumour cells to support biosynthesis (173). Hexokinase-2 (Hk2) is a key metabolic regulator induced by SHH, its importance is highlighted upon deletion, which leads to impairment in CGCP development and reduced tumorigenesis in the MB-prone SmoM2 mouse model (174). The nutrient sensor peroxisome proliferator-activated receptor γ (PPARγ) is also involved in SHH-mediated regulation of glycolysis; pharmacological blockade of PPARγ inhibits CGCP proliferation and extends animal survival in the NeuroD2-SmoA1 mouse model of MB by inducing cell death (170).

The activation of *MYC* family is a key point of convergence of the metabolic features of many different cancer types. Similar to its family members, MYCN is a potent regulator of cellular metabolism, through controlled expression of amino acid transporters and other proteins involved in aerobic glycolysis, oxidative phosphorylation, detoxification of reactive oxygen species (ROS), and fatty acid oxidation (175). While numerous studies have demonstrated a key role of MYCN in NB and GBM metabolism (176–180), its metabolic function in medulloblastoma still remains elusive. It is likely that in these tumours, as in other cancers, MYCN reconfigures metabolism to favour aerobic glycolysis and a dependency on the serine-glycine-one-carbon (SGOC) to generate metabolic products starting from serine and glycine amino acids (181).

**Figure 3:**
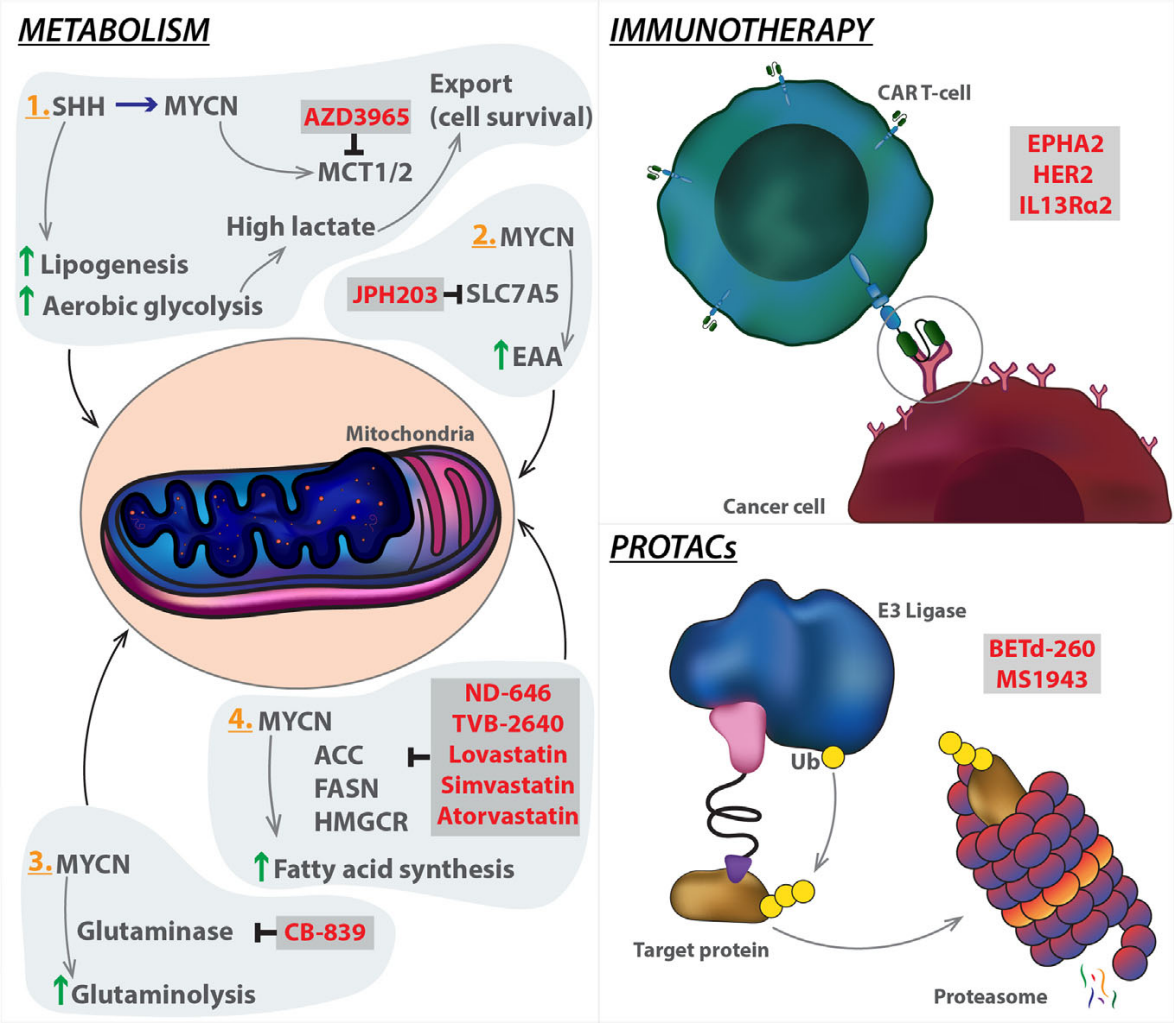
Emerging strategies targeting MYCN. Innate systems regulating metabolism and immune response can be manipulated in cancer models to hijack the tumorigenic mechanism. PROTAC technology can be used to target MYCN (the target protein) by enhancing protein degradation through coupling with E3 ligase, which ubiquitinates the protein leading to degradation via the proteasome. Specific modalities (highlighted in red) have been developed to target the mechanistic components of each pathway. SHH, Sonic hedgehog; MCT1/2, Monocarboxylate transporter 1; SLC7A5, Solute carrier family 7 member 5; EAA, Essential amino acid; CAR T-cell, Chimeric antigen receptor T cells; ACC, Acetyl-CoA carboxylase; FASN, Fatty acid synthase; HMGCR, 3-hydroxy-3-methylglutaryl-CoA reductase; PROTACs, proteolysis targeting chimeras.

Selective targeting of tumour glucose metabolism has long been considered as an attractive therapeutic strategy. MYC invariably promotes expression of critical enzymes involved in aerobic glycolysis, such as HK2 and LDHA, making cancer cells more vulnerable to glycolysis inhibition. 2-Deoxyglucose, an analogue of glucose that binds and inhibits HK2, has yielded promising antitumour activity *in vitro* and *in vivo*. Aerobic glycolysis produces excessive lactate that is toxic to tumour cells. MYC modulates lactate export by inducing MCT1/MCT2 expression to shift toxic levels of lactate within tumour cells. Therefore, a potential, effective strategy is to block MYC-driven lactate export by MCT1/MCT2 inhibitors. Of note, clinical trials of the MCT1 inhibitor AZD3965 in diffuse large B cell lymphoma and Burkitt's lymphoma, two typical MYC-driven cancer types, are currently ongoing [NCT01791595] (**Figure 3** **)**.

Inhibitors of glutaminase or transaminase have shown the therapeutic efficacy in multiple MYC-driven tumour models, and a representative glutaminase inhibitor, CB-839, is currently under clinical trials for patient treatment. MYC and SLC7A5 constitute a feedback loop to amplify MYC transcriptional program, and sustain essential amino acid (EAA) metabolism in tumour cells (182). In principle, therapeutic targeting of SLC7A5 would offer an opportunity to unleash the functional association between MYC and SLC7A5, leading to tumour suppression. JPH203 (also known as KYT-0353), a specific SLC7A5 inhibitor (183) can be evaluated as a MYC-selective cancer therapeutics in the future clinical trials (**Figure 3** **)**. MYC is a key player in regulation of lipid metabolic reprogramming. ACC, FASN, and 3-Hydroxy-3-Methylglutaryl-CoA reductase (HMGCR), three key enzymes for lipid metabolism, are significantly activated by MYC. ND-646, an allosteric inhibitor of ACC that prevents ACC dimerization and subsequently suppresses fatty acid synthesis, has shown efficacy in mouse models of lung cancer (184). TVB-2640 is a highly potent, selective, and reversible first-in-class inhibitor of FASN. Its monotherapy and in combination with paclitaxel have entered the clinical trial stage [NCT03179904]. Lovastatin, simvastatin, and atorvastatin are specific HMGCR inhibitors that have been FDA approved to lower cholesterol (185). Targeting these enzymes may be a therapeutic alternative for MYC-driven cancers (**Figure 3** **)**. However, caution should also be taken because it remains unclear as to which aspects of cell metabolism could represent a realistic, targetable vulnerability of tumour cells in comparison with normal counterparts. It should be noted that cancer cells acquire metabolic adaptations in response to a variety of cell-extrinsic and cell-intrinsic cues, thus, MYC effects on cellular metabolism depend both on the tissue of tumour origin and on interaction with tumour microenvironment. A better understanding of these metabolic diversities will improve our ability to define their contribution to aggressive tumour progression.

### Immunotherapy in Medulloblastoma

The last decade has seen a tremendous progress in our understanding of how cancer cell evade the immune system and how to harness these mechanisms to develop new therapies. Cancer immunotherapy has proven successful in the so-called "hot" tumours, such as lung cancer and melanoma, characterized by high infiltrating immune cells, while "cold" tumour with low infiltrates still represents a therapeutic challenge for immunotherapy.

The MB microenvironment inhabits reduced numbers of infiltrating immune cells and have been generally considered as immunologically "cold". This is largely backed by a limited amount of information existing on the immune microenvironment. Whilst this is the current understanding, the paracrine signalling between the tumour microenvironment suggests the existence of a more intricate interaction (186). More recently, an increasing number of studies have shed light on the immune profiling of MB, in an attempt to use these data as diagnostic and prognostic tools. Grabovska and colleagues have mapped the tumour immune microenvironment of >6000 primarily paediatric tumour of the CNS, by using methylCIBERSORT, an algorithm derived from CIBERSORT and based on genome-wide DNA methylation data (187, 188). By combining methyCIBERSORT data with pre-existing clinico-pathological and parallel multi-omics, the study exhibited varying proportions of infiltrates within the four classic subgroups of MB. CD8+ T cells (27% of all non-cancer cells), B cells (16%), and eosinophils (15%) (187) were the most abundantly estimated non-cancer cell infiltrates within all MB tumours. The distribution of cell types within the subgroups showed Grp3 MB holding the highest proportion of CD8+ T cells, Grp4 MB homing the natural killer (NK) cells, and SHH MB, the B cells. MYC amplification in Grp3 MB is associated with a significantly higher frequency of tumour infiltrating lymphocytes, CD8+ T cells, and B cells and a lower infiltration of regulatory T cells (Treg). Interestingly, this immune infiltrate analysis further supports the recent refinement of the Grp3/Grp4 MB subgroups into eight subtypes I–VIII (83).

In all cases, the methyCIBERSORT estimates of TILs aligns with the "Cytolytic score", derived from the expression of granzyme A (GZMA) and perforin 1 (PRF1), secreted by effector cytotoxic T cells and NK cells. In another gene expression study, a smaller cohort of SHH MB tumours show high content of fibroblasts, T cells and macrophages, whilst Grp4 MB expresses markers of cytotoxic lymphocytes. SHH MB subgroup has increased expression of inflammation-related genes (CD14, PTX3, CD4, CD163, CSF1R, and TGFB2) and significantly higher infiltration of tumour-associated fibroblasts than Grp3 MB and Grp4 MB (189). In another cohort, cytotoxic T-cells, with variable activation status, showed no correlation with overall survival of the patients (190). While these studies prove that immune profiles are specific to the different molecular subgroups of MB, their applicability in the clinical settings is still unclear. Moreover, data on the immune checkpoint proteins, PD-1 and PD-L1 are limited and controversial, due both to technical challenges to detect these markers or discrepancy between *in vitro* and *in vivo* results (191–193).

Another reason why MB are considered "cold" is the relatively low expression of cancer-specific antigens on their cell surface. Orlando and colleagues have recently reported expression of the tumour-associated antigen PRAME in 82% of MB tumour tissues. However, its levels only showed correlation with the worst overall survival groups. Moreover, MB cells targeted using genetically modified T cells carrying a PRAME-specific TCR controlled tumour growth in an orthotopic mouse model of MB (194). Intrathecal delivery of T cells engineered to express EPHA2, HER2 and interleukin 13 receptor α2 (IL13Rα2) chimeric antigen receptors showed efficacy in the treatment for primary, metastatic, and recurrent Grp3-MB xenografts in mouse models. Administration of these chimeric antigen receptor T (CAR-T) cells into the CSF, alone or in combination with the epigenetic modifier azacytidine, was highly effective against different metastatic mouse models of Grp3 MB, thereby providing a rationale for CAR-T approaches in the clinic (195) **(** **Figure 3** **)**.

While computational analyses are advancing our general knowledge of MB-tumour microenvironment (TME), the direct link between MYCN expression and TME profiling is still under investigation. What has emerged in neuroblastoma and other malignancies (small cell lung cancer, rhabdomyosarcoma, Wilms' tumour, retinoblastoma, acute myeloid leukaemia, and T-acute lymphoid leukaemia) is that MYCN has a great role in dysregulating the immune network. In neuroblastoma patients for instance, gene set enrichment analysis has shown that *MYCN* levels negatively correlate with genes involved in different immune system pathways, especially those associated to interferon gamma and phagocytosis (196). Overall, MYCN suppresses the immune landscape, through dysregulation of immune checkpoints, CD4+ helper T (Th) cytokines, major histocompatibility complex (MHC) genes, and Toll-like receptors (TLRs) (197). Apart from tumour cases with MYCN gene amplification, the immune system dysregulation can occur as a consequence of other events leading to increased MYCN activity (mRNA and protein stabilization, mi-RNA alteration). Based on these considerations, it is imperative to have a better understanding of the mechanistic components linking MYCN to MB-TME functions. The blockage of MYCN or specific MYCN dependencies could ameliorate the immune suppression by restoring the responsiveness of the immune system, opening the way to combinatorial treatments with immunotherapies. In this context, the link between MYCN and polycomb repressive complex 2 (PRC2) may offer a promising therapeutic opportunity via a mechanism that alters TME immunogenicity (198, 199).

### Use of PROTACs

Proteolysis targeting chimeras (PROTAC) and hydrophobic tagging are successful technologies/strategies for selective degradation of the target protein (200, 201). Although PROTAC technology has been rapidly gaining momentum in the drug discovery field, the hydrophobic tagging approach has received considerably less attention from the biomedical community. This approach utilizes a bulky and hydrophobic group attaching to a small-molecule binder of the target protein. The binding of this bivalent compound to the target protein leads to misfolding of the target protein and its subsequent degradation by the proteasome (202). Targeting oncogenic proteins for degradation using PROTACs recently gained an increased momentum in the field of cancer research. Compared with BET inhibitors HJB-97 and JQ1, the activity of the PROTAC BET degrader BETd-260 increased over 1000 times (203). The degrader complex showed stability through cooperative binding between AURKA and CEREBLON (204). The enhancer of zeste homolog 2 (EZH2) is the main enzymatic subunit of the polycomb repressive complex 2, which catalyses tri-methylation of lysine 27 on histone H3 (H3K27me3) to promote transcriptional silencing. PRC2 complex has important roles in tissue development, primarily to maintain cell identity (205). EZH2 is overexpressed in multiple types of cancer including triple-negative breast cancer (TNBC), and high expression levels correlate with poor prognosis. In MB, studies have shown increased expression of EZH2 in all subgroups, with particularly high levels in G3 and G4 (206, 207). The link between this enzyme and MYC or MYCN remains largely unexplored. Whilst studies have shown correlation between levels of the enzyme and MYC activity (208), the causation behind this is yet to be mapped. Nonetheless, Chen et al. showed in MYCN amplified neuroblastoma, a strong dependency between tumour cells and the PRC2 complex. MYCN was shown to directly activate EZH2 by binding to its promoter, leading to inhibition of neuronal differentiation networks in *MYCN*-amplified (209). This approach should also be applied to MB to elucidate the underlying mechanism. Several EZH2 inhibitors, which inhibit the methyltransferase activity of EZH2, have shown promising results in treating sarcoma and follicular lymphoma in clinics. However, EZH2 inhibitors are ineffective at blocking proliferation of TNBC cells, even though they effectively reduce the H3K27me3 mark. Using a hydrophobic tagging approach, generation of MS1943, a first-in-class EZH2 selective degrader that effectively reduces EZH2 levels in cells (210) **(** **Figure 3** **)**.

## Conclusion and Future Outlook

In this review, we have highlighted the relationship between *MYCN* and the paediatric brain tumour medulloblastoma, with an emphasis on the emerging therapeutic avenues to target this. The ever-increasing advancements in sequencing technologies, coupled with global efforts to improve the disease models through strong collaborations, and the use of more humanised systems, is rapidly dissecting the precise role of MYCN in all MB subgroups. Model systems such as patient-derived iPSCs (51, 211, 212), and human hindbrain-derived neuroepithelial stem cells (213) align with the developmental trajectory of the CNS, therefore are likely to reflect a more authentic evolution of the tumour through targeting of relevant oncogenes such as MYCN. Novel MB targeting strategies using PROTACs, and CAR T-cell therapy offer a selective advantage over the more generic inhibitors. Additional focus on the metabolic dependencies of MB tumours can shed light on the most vulnerable target for tumour growth. Whilst the supporting evidence indicate a practical use for these technologies in MB, these mechanisms remain largely unexplored. Recently, liquid biopsies using CSF to assess circulating tumour DNA have been used to genetically characterise MB (214). This is an important development in the management of MB, one particularly relevant to the potential MYCN-focussed therapeutic approaches discussed here, as liquid biopsies detect the majority of MYCN-effecting mutations such as *MYCN* and *Gli2* amplification, and *SUFU* loss (214). As CSF may be obtained during hydrocephalus surgery, a common procedure for MB patients, this will open the possibility to personalised medicine approaches for the treatment of this devastating disease. Furthermore, MB is a brain tumour protected from the systematic delivery of cancer drugs by the blood brain barrier (BBB). This defence system is a unique problem which prevents majority of the current therapies from succeeding. Utilising lipid-soluble cargoes such as nanoparticles which disintegrate at the target site (215, 216), or focused ultrasound techniques e.g., pulsed ultrasound (217), can greatly improve the delivery of targeted drugs.

It is certainly evident that MYCN is a phenomenally complex molecule. As illustrated in this review, the multiple downstream signalling pathways directly or indirectly regulated by Myc highlights that targeting this oncogene is a compelling, yet challenging strategy for MB. Our ultimate goal is to increase the proportion of surviving patients, more specifically by reverting the adverse effects of disseminated disease and treatment sequelae. Thus, our expanding knowledge of the mechanisms in this cancer offers the promise to formulate more targeted therapies and translate this to the clinic in the best form.

## Author Contributions

SS and CG wrote the review with advice and contribution from AM and LC. SS made the figures with input from AM and CG. All authors contributed to the article and approved the submitted version.

## Funding

This review article was financially supported by The Institute of Cancer Research PhD studentship (SS), Higher Education Funding Council of England (LC) and The Institute of Cancer Research funding scheme (CG).

## Conflict of Interest

The authors declare that the research was conducted in the absence of any commercial or financial relationships that could be construed as a potential conflict of interest.
